# The relationship of apolipoprotein B and very low density lipoprotein triglyceride with hyperuricemia and gout

**DOI:** 10.1186/s13075-014-0495-z

**Published:** 2014-11-29

**Authors:** Humaira Rasheed, Angela Hsu, Nicola Dalbeth, Lisa K Stamp, Sally McCormick, Tony R Merriman

**Affiliations:** Department of Biochemistry, University of Otago, 710 Cumberland Street, Dunedin, 9052 New Zealand; Department of Chemistry, University of Engineering and Technology, G.T. Road, Lahore, 54890 Pakistan; Department of Medicine, University of Auckland, Park Road, Auckland, 1010 New Zealand; Department of Medicine, University of Otago, 2 Riccarton Avenue, Christchurch, 8140 New Zealand

## Abstract

**Introduction:**

Gout results from an innate immune response to monosodium urate (MSU) crystals deposited in joints. Increased very low-density lipoprotein (VLDL) has been associated with gout. The apolipoprotein B (apo B), which is present on VLDL, regulates neutrophil response to MSU crystals and has been positively associated with gout. Furthermore, the gene (*A1CF*) encoding the complementation factor for the *APOB* mRNA-editing enzyme is associated with urate levels. However, the relationship of apo B and VLDL with gout and hyperuricaemia (HU) is still unclear. Therefore, we tested the association of VLDL and apo B with HU and with gout compared to HU.

**Methods:**

New Zealand European (n = 90) and Māori and Pacific Island (Polynesian) (n = 90) male gout case and control sample sets were divided into normouricaemia (NU), asymptomatic HU and gout groups. Size exclusion chromatography and enzyme-linked immunosorbant assay was used to measure VLDL and apo B. Multivariate logistic regression was used to assess the risk of gout and HU per unit change in VLDL and apo B.

**Results:**

Increased levels of VLDL triglycerides (Tg) were observed in the gout sample set compared to NU and HU in Europeans (*P* = 1.8 × 10^-6^ and 1 × 10^-3^, respectively), but only compared to NU in Polynesians (*P* = 0.023). This increase was driven by increased number of VLDL particles in the European participants and by the Tg-enrichment of existing VLDL particles in the Polynesian participants. Each mmol/L increase in VLDL Tg was significantly associated with gout in the presence of HU in Europeans, with a similar trend in Polynesians (OR = 7.61, *P* = 0.011 and 2.84, *P* = 0.069, respectively). Each μmol/L increase in total apo B trended towards decreased risk of HU (OR = 0.47; *P* = 0.062) and, conversely, with increased risk of gout compared to HU (OR = 5.60; *P* = 0.004).

**Conclusions:**

Increased VLDL Tg is associated with the risk of gout compared to HU. A genetic approach should be taken to investigate the possibility for causality of VLDL in gout. Apolipoprotein B may have pleiotropic effects in determining HU and gout.

## Introduction

Gout is caused by activation of the innate immune system in response to monosodium urate (MSU) crystals that are deposited in joints when serum urate levels are elevated. The genetic and environmental causal factors in determining hyperuricaemia (HU) are becoming better understood [1,2]. However, causal mechanisms in determining gout in the presence of HU are poorly understood. Along with chylomicrons, very low-density lipoproteins (VLDL) are the lipoproteins that primarily transport triglycerides (Tg) with 86% and 55% of their core lipids constituting Tg, respectively. Gout has been associated with increased levels of VLDL Tg in a non-obese and non-drinking male Japanese sample set [3] and type IV hyperlipoproteinaemia (characterized by increased VLDL-Tg) is prevalent in Japanese male gout patients (39%) [3]. The increased VLDL Tg levels in gout could be caused, at least in part, by reduced lipoprotein lipase activity [4]. Apolipoprotein B (apo B), amongst other lipoproteins, coats MSU crystals exposed to plasma and, when attached to MSU crystals as part of an entire lipoprotein particle, can suppress the stimulation of neutrophils [5]. Based on their previous observation of elevated VLDL components and increased ratio of apolipoprotein CIII (involved in VLDL clearance) to CII in hyperuricaemic-hypertriglyceridaemic patients, Cardona *et al*. [6] demonstrated association of genetic variants at the APOCIII locus with gout. Also, increased VLDL Tg is associated with reduced urinary uric acid excretion [3,7]. Elevated serum apo B levels have also been associated with primary gout [8]. Interestingly, there is association between urate levels and risk of gout of the gene (*A1CF*), which encodes the complementation factor for the APOBEC enzyme that edits apo B mRNA [9,10].

Collectively the observations outlined above implicate VLDL Tg and/or apo B in gout. However, the relationship between VLDL-Tg, apo B and HU and gout is unclear. We hypothesized that if increased VLDL Tg plays a role in regulation of the innate immune response to MSU crystals in gout then this would be consistent with association of VLDL Tg with gout compared to HU. We also tested for association of circulating levels of apo B with HU and gout compared to HU.

## Methods

### Participants

The 180 New Zealand (NZ) male participants consisted of gout cases, defined by the American Rheumatology Association preliminary classification criteria [11], and controls, who self-reported their lack of gouty arthritis and were at least 17 years of age. Gout cases were recruited from the Auckland and Canterbury regions of NZ. Participants without gout self-reported no diagnosis of gout, and were convenience sampled from the Auckland and Otago regions of NZ. Control participants were stratified into a normouricaemia (NU) group with serum urate level of <0.41 mmol/L and a HU group with serum urate level ≥0.41 mmol/L. All variables except for biochemical measurements, body mass index (BMI) and medications (obtained from medical records) were self-reported. Ancestry was self-reported and participants were divided into NZ Europeans and NZ Polynesians (Māori and Pacific Island). Demographic, anthropomorphic and clinical data are reported in Table 1. The New Zealand Multi-region Ethics Committee (MEC/105/10/130) approved the study and all participants gave written informed consent.

**Table 1:** **Characteristics of the sample sets**

	**Polynesians**			***P*** **values**		
	**NU**	**HU**	**Gout**	**NU vs. HU**	**NU vs. Gout**	**HU vs. Gout**
Number	30	30	30	30	30	30
Age (year)	46.07 ± 12.18	45.83 ± 12.32	42.43 ± 9.95	0.94	0.21	0.24
Waist circumference (cm)	105.72 ± 11.03	111.31 ± 15.32	110.00 ± 11.20	0.12	0.15	0.71
BMI (kg/cm2)	32.97 ± 5.60	34.68 ± 6.06	34.52 ± 5.31	0.27	0.28	0.92
eGFR (mL/min/1.73 m^2^)	83.03 ± 16.14	72.30 ± 12.35	74.77 ± 15.36	0.0054	0.047	0.50
Hypertension (%)	17.24	16.67	26.67	0.95	0.38	0.35
Type 2 diabetes (%)	13.33	6.67	6.9	0.39	0.41	0.97
Statin use (%)	13.33	13.33	30.00	0.96	0.20	0.22
Fibrate use (%)	_	_	3.33	_	_	_
Tophi (%)	_	_	53.33	_	_	_
Allopurinol use (%)	_	_	68.97	_	_	_
Probenecid usage (%)	_	_	7.41	_	_	_
Fruit consumption (pieces per day)	2.62 ± 1.37	1.88 ± 1.46	1.23 ± 0.96	0.051	<0.00001	0.049
Sugary drink consumption (drinks per day)	2.41 ± 2.40	2.06 ± 1.91	2.78 ± 2.07	0.54	0.53	0.17
Alcohol consumption (drinks/week)	2.40 ± 6.02	4.23 ± 7.95	8.87 ± 13.53	0.32	0.020	0.11
Serum urate (mmol/L)^1^	0.346 ± 0.040	0.474 ± 0.063	0.469 ± 0.107	<0.00001	<0.00001	0.84
		**Europeans**			***P*** **values**	
	**NU**	**HU**	**Gout**	**NU vs. HU**	**NU vs. Gout**	**HU vs. Gout**
Number	30	30	30			
Age (year)	57.67 ± 12.01	63.17 ± 13.61	53.60 ± 9.11	0.10	0.14	0.0022
Waist circumference (cm)	92.25 ± 15.08	99.38 ± 13.53	107.79 ± 9.92	0.090	<0.00001	0.0096
BMI (kg/cm2)	25.73 ± 6.53	28.85 ± 6.88	31.27 ± 4.18	0.12	0.0001	0.11
eGFR (mL/min/1.73 m^2^)	80.18 ± 12.49	65.91 ± 16.25	68.86 ± 17.47	0.0004	0.0059	0.50
Hypertension (%)	23.33	36.67	46.67	0.26	0.058	0.43
Type 2 diabetes (%)	3.45	6.9	3.33	0.55	0.98	0.53
Statin use (%)	20.00	23.33	16.67	0.75	0.74	0.52
Fibrate use (%)	_	_	3.33	_	_	_
Tophi (%)	_	_	13.79	_	_	_
Allopurinol use (%)	_	_	75.86	_	_	_
Probenecid usage (%)	_	_	3.7	_	_	_
Fruit consumption (pieces per day)	1.68 ± 1.09	2.27 ± 1.62	2.00 ± 1.39	0.11	0.33	0.50
Sugary drink consumption (drinks per day)	0.77 ± 1.27	1.42 ± 1.77	0.69 ± 1.04	0.11	0.79	0.060
Alcohol consumption (drinks/week)	5.40 ± 6.13	8.68 ± 14.72	6.53 ± 6.06	0.26	0.47	0.46
Serum urate (mmol/L)	0.321 ± 0.046	0.459 ± 0.041	0.403 ± 0.094	<0.00001	0.0001	0.0042

^1^In the European gout cases, 17 were HU and 13 NU at the time of recruitment. In the Polynesian cases, the corresponding numbers were 22 and 8, respectively. NU: normouricaemia; HU: hyperuricaemia; BMI: body mass index; eGRF: estimated glomerular filtration rate.

### Biochemical measurements

Fast protein liquid chromatography (FPLC) was used to fractionate lipoproteins from frozen non-fasting serum samples. Tg and cholesterol assays were done on the FPLC fractions to identify lipoprotein-containing fractions. Dilutions of Precipath L (Roche, Mannheim, Germany) were used to make a standard curve and all samples were tested in triplicate. A total of 100 μL of each fraction was incubated with 100 μL of Tg glycerol-3-phosphate oxidase-phenol and aminophenazone (GPO-PAP) reagent (Roche/Hitachi) or cholesterol oxidase (CHOD)-PAP reagent (Roche) for 15 to 20 minutes at 37°C in a 96-well plate (Nunc™, Thermo Fisher Scientific, Waltham, MA, USA). Non-fasting serum samples were studied because participants were convenience sampled from health care and community settings.

An enzyme-linked immunosorbant assay (ELISA) was used to measure apo B in serum and FPLC fractions as previously described [12]. Cfas Lipid (Roche) with a known apo B concentration of 2,185 nmol/L was used as an internal control. The primary antibody used for apo B measurement was a polyclonal antibody (Roche) and the secondary antibody was an anti-sheep immunoglobulin G (IgG) conjugated to horseradish peroxidase. Total apo B values obtained from ELISA were adjusted for the percentage lipid recovery from the FPLC. This was done for each individual sample by multiplying the total apo B value by the percentage recovery of plasma lipids from the FPLC. Serum urate was measured by the uricase oxidation method [13] and serum creatinine was measured using the creatinine Jaffé compensated method [14], the end point determined by a Roche Cobas™ 8000 analyser. The estimated glomerular filtration rate (eGFR) was calculated using the Modification of Diet in Renal Disease formula: eGFR (mL/min/1.73 m^2^) = 175 × ((SCr × 0.0113)^-1.154^ × (Age^-0.203^).

### Statistical analyses

Due to the skewed distribution of lipid-related phenotypes, all lipid, lipoprotein and apolipoprotein values were expressed as median and interquartile range (25th to 75th percentile) and pairwise comparisons were done using the Wilcoxon-Mann-Whitney test or Student *t* test or chi-square test via the Intercooled STATA™ software version 8.0 (StataCorp, College Station, TX, USA). Multivariate-adjusted linear and logistic regression analyses were used to assess the association of VLDL and other lipid variables with phenotype. A threshold of *P* <0.05 was used to indicate statistical significance.

## Results

**Figure 1:**
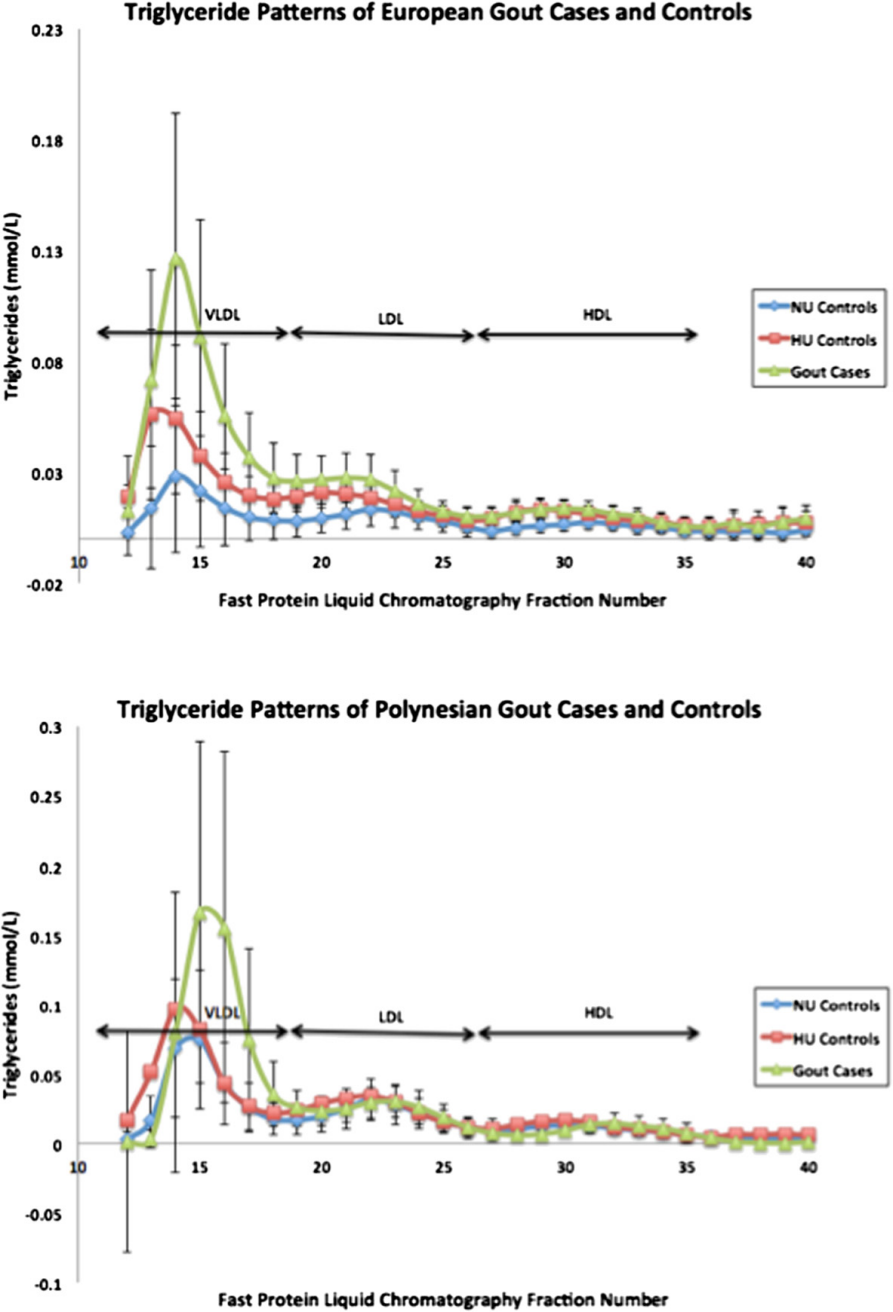
**Average triglyceride lipoprotein traces of gout cases and controls.** Lipoproteins in plasma samples were separated by fast protein liquid chromatography and each fraction was assayed for triglyceride concentration.

Traces of Tg concentrations in FPLC fractionated lipoproteins (Figure 1) indicate differences in Tg content in VLDL fractions between the gout and non-gout groups in both Europeans and Polynesians. An increasing trend in serum, total FPLC Tg and VLDL Tg from NU to HU to gout was observed in both Europeans and Polynesians (Table 2). VLDL Tg was significantly higher in gout than NU and HU in Europeans (Table 2; *P* = 1.84 × 10^-6^ and 0.001, respectively) whereas the difference was significant between NU and gout in Polynesians (Table 2; *P* = 0.023). FPLC is a standard methodology used to separate lipoproteins by size [15]. The area under the curve of the resulting lipoprotein peaks as identified by cholesterol or triglyceride assay can be utilised to establish ratios of the three major lipoprotein fractions that is VLDL, low-density lipoprotein (LDL) and high-density lipoprotein (HDL). Therefore lipid ratios rather than absolute lipid values were used for further analyses. Significant differences were found in the VLDL Tg to total Tg ratio between European gout cases and HU and NU in the three pairwise comparisons (Table 2; *P* = 0.0041 to 1.23 × 10^-06^), with the ratio increasing from NU to HU to gout. However, in the Polynesian groups, this ratio was not significantly different between NU and HU controls (*P* = 0.44) but was significantly increased in gout compared to NU and HU (*P* = 0.0071 and 0.0011 respectively).

**Table 2:** **Fast protein liquid chromatography lipid measurements**

	**Polynesians**					
	**NU median [25th-75th percentile]**	**HU median [25th-75th percentile]**	**Gout median [25th-75th percentile]**	***P*** **NU vs. HU**	***P*** **NU vs. Gout**	***P*** **HU vs. Gout**
Number	30	30	30			
Total serum cholesterol	5.71 [4.33-6.47]	5.73 [5.03-6.54]	5.99 [5.25-6.41]	0.48	0.47	0.86
Total FPLC cholesterol	4.75 [3.52-5.47]	4.16 [3.72-4.81]	4.00 [3.18-4.43]	0.25	0.074	0.41
LDL cholesterol	2.83 [2.20-3.22]	2.45 [2.14-3.09]	2.45 [1.99-2.97]	0.42	0.11	0.40
HDL cholesterol	1.13 [0.84-1.35]	0.96 [0.79-1.00]	0.77 [0.70-1.06]	0.030	0.0052	0.17
Total serum Tg	1.87 [1.15-3.04]	2.32 [1.83-3.29]	2.49 [1.46-4.36]	0.058	0.060	0.93
Total FPLC Tg	1.41 [0.97-2.16]	1.55 [1.23-1.80]	1.71 [1.09-2.85]	0.34	0.17	0.46
VLDL Tg	0.57 [0.37-1.18]	0.80 [0.61-1.06]	1.01 [0.60-2.11]	0.24	0.023	0.10
VLDL Tg:Total Tg	0.45 [0.32-0.61]	0.51 [0.43-0.60]	0.65 [0.53-0.75]	0.44	0.0071	0.0011
Total apo B (μmol/L)	1.09 [0.93-1.85]	0.94 [0.79-1.13]	1.10 [0.92-1.26]	0.026	0.59	0.020
VLDL apo B (μmol/L)	0.044 [0.028-0.079]	0.072 [0.045-0.101]	0.061 [0.028-0.132]	0.048	0.32	0.92
VLDL apo B:Total apo B	0.040 [0.025-0.077]	0.072 [0.050-0.11]	0.051 [0.022-0.121]	0.001	0.19	0.40
VLDL Tg:VLDL apo B	12.49 [7.95-20.14]	11.74 [8.23-15.26]	19.44 [13.61-25.83]	0.74	0.042	0.0011
			**Europeans**			
Number	30	30	30			
Total serum cholesterol	5.56 [4.75-6.09]	5.60 [4.93-6.39]	5.11 [4.27-6.59]	0.77	0.37	0.36
Total FPLC cholesterol	4.18 [3.73-4.86]	4.01 [3.19-4.70]	4.19 [3.28-5.10]	0.21	0.70	0.50
LDL cholesterol	2.51 [2.06-2.97]	2.45 [1.86-2.83]	2.37 [1.83-3.59	0.46	0.68	0.75
HDL cholesterol	1.27 [1.21-1.57]	1.01 [0.79-1.21]	1.00 [0.89-1.10]	2.14E-05	0.0001	0.79
Total serum Tg	1.58 [1.05-2.00]	1.92 [1.50-2.80]	2.45 [1.70-2.90]	0.019	0.0008	0.21
Total FPLC Tg	1.11 [0.64-1.35]	1.15 [0.90-1.41]	1.57 [1.23-2.25]	0.17	0.0001	0.0021
VLDL Tg	0.36 [0.16-0.47]	0.47 [0.35-0.65]	0.84 [0.59-1.30]	0.008	1.84E-06	0.0010
VLDL Tg:Total Tg	0.32 [0.24-0.41]	0.43 [0.35-0.50]	0.68 [0.49-0.60]	0.0041	1.23E-06	0.0004
Total apo B (μmol/L)	1.31 [1.09-1.65]	1.06 [0.72-1.42]	1.36 [1.03-1.76]	0.036	0.88	0.053
VLDL apo B (μmol/L)	0.038 [0.021-0.072]	0.064 [0.035-0.103]	0.090 [0.062-0.125]	0.018	4.53E-05	0.041
VLDL apo B:Total apo B	0.032 [0.019-0.054]	0.063 [0.043-0.090]	0.069 [0.051-0.092]	0.0001	8.59E-06	0.37
VLDL Tg:VLDL apo B	8.33 [4.76-13.61]	8.62 [5.74-12.56]	10.45 [7.41-13.72]	0.92	0.16	0.16

All *P* values are calculated using the Wilcoxon-Mann-Whitney test. NU: normouricaemia; HU: hyperuricaemia; FPLC: fast protein liquid chromatography; LDL: low-density lipoprotein; HDL: high-density lipoprotein; Tg: triglyceride; VLDL: very low-density lipoprotein; apo B: apolipoprotein B.

The VLDL apo B concentration was significantly higher in HU and gout cases in Europeans compared to NU (Table 2; *P* = 0.018 and 4.5x10^-5^, respectively) and in gout compared to HU (*P* = 0.041). In the Polynesian group the corresponding value for VLDL apo B concentration was not significantly different between gout and the other subgroups, although it was higher in gout. There was a significant increase in the VLDL apo B to total apo B ratio in HU and gout compared to NU in Europeans and between NU and HU in Polynesians (Table 2). There was no significant difference in the VLDL Tg to VLDL apo B ratio between European subgroups; conversely, in Polynesians, this ratio was higher in gout compared to HU and NU (Table 2; *P* = 0.0011 and 0.042, respectively). Collectively these results suggested an increased number of VLDL particles in HU particularly in Europeans, with an increase in Tg loading of these particles evidenced in gout in Polynesian participants.

We tested the risk of gout compared to NU and HU for three Tg phenotypes (Table 3; total serum Tg; total FPLC Tg, VLDL Tg). In the presence of HU each mmol/L increase in VLDL Tg associated with a 7.61-fold increase in risk in Europeans (*P* = 0.011) and a trend towards increased risk in Polynesians (odds ratio (OR) = 2.84; *P* = 0.069), with significantly increased risk in the combined ancestral groups (OR = 3.56; *P* = 0.002). These results were robust to exclusion of gout cases taking fenofibrates or statins (Table 4). A significant effect was seen for total FPLC Tg in Europeans comparing gout to HU (OR = 5.19 per unit mmol/L increase, *P* = 0.014) but not Polynesians (OR = 1.52, *P* = 0.31). There was no significant risk in gout associated with total serum Tg.

**Table 3:** **Analysis of association of gout risk with lipid and apo B-associated traits in study sample sets**

	**NU vs. Gout**				**HU vs. Gout**			
	**Unadjusted OR [95% CI]**	***P***	**Adjusted OR [95% CI]**	***P***	**Unadjusted OR [95% CI]**	***P***	**Adjusted OR [95% CI]**	***P***
**Total Tg (mmol/L)**								
Europeans	2.34 [1.22-4.49]	0.011	1.37 [0.70-2.65]	0.36	1.21 [0.73-1.99]	0.46	1.31 [0.72-2.40]	0.38
Polynesians	1.41 [1.00-1.98]	0.049	1.00 [0.51-1.98]	0.99	1.17 [0.85-1.62]	0.34	1.14 [0.75-1.75]	0.54
Combined	1.59 [1.17-2.17]	0.003	1.19 [0.80-1.75]	0.39	1.18 [0.90-1.54]	0.24	1.20 [0.88-1.65]	0.25
**Total FPLC Tg (mmol/L)**								
Europeans	4.17 [1.51-11.51]	0.006	1.97 [0.75-5.15]	0.17	4.37 [1.49-12.81]	0.007	5.19 [1.39-19.33]	0.014
Polynesians	1.29 [0.80-2.10]	0.30	0.65 [0.28-1.50]	0.31	1.51 [0.82-2.78]	0.19	1.52 [0.68-3.41]	0.31
Combined	1.74 [1.14-2.64]	0.01	1.17 [0.69-1.96]	0.56	2.05 [1.23-3.42]	0.006	2.14 [1.17-3.90]	0.014
**VLDL Tg (mmol/L)**								
Europeans	11.29 [2.40-53.00]	0.002	3.24 [0.87-12.07]	0.080	6.52 [1.70-24.99]	0.006	7.61 [1.60-36.26]	0.011
Polynesians	1.92 [1.01-3.67]	0.048	0.98 [0.34-2.81]	0.97	2.46 [1.11-5.44]	0.026	2.84 [0.92-8.76]	0.069
Combined	2.78 [1.55-4.99]	0.001	1.81 [0.87-3.75]	0.11	3.19 [1.62-6.24]	0.001	3.56 [1.60-7.92]	0.002
**Total apoB (** **μmol/L)**								
Europeans	0.97 [0.36-2.63]	0.95	0.83 [0.18-3.76]	0.81	2.71 [0.94-7.76]	0.064	3.32 [0.87-12.61]	0.078
Polynesians	0.47 [0.18-1.23]	0.12	0.27 [0.04-1.73]	0.17	13.38 [1.48-120.76]	0.021	33.55 [1.16-972.93]	0.041
Combined	0.65 [0.34-1.26]	0.20	0.78 [0.32-1.93]	0.59	3.60 [1.41-9.17]	0.007	5.60 [1.73-18.18]	0.004
**VLDL apo B (0.1** **μmol/L)**								
Europeans	17.80 [2.99-106.02]	0.002	10.05 [1.16-87.35]	0.036	1.69 [0.74-3.86]	0.21	1.67 [0.62-4.51]	0.31
Polynesians	2.24 [0.89-5.60]	0.086	1.97 [0.48-8.12]	0.35	1.51 [0.61-3.73]	0.38	2.42 [0.70-8.36]	0.16
Combined	4.23 [1.87-9.59]	0.001	2.68 [1.03-6.95]	0.043	1.60 [0.87-2.93]	0.13	1.57 [0.80-3.09]	0.19
**VLDL Tg:VLDL apo B**								
Europeans	1.06 [0.97-1.16]	0.20	1.02 [0.90-1.16]	0.78	1.06 [0.97-1.17]	0.20	1.02 [0.91-1.15]	0.68
Polynesians	1.05 [0.99-1.10]	0.086	1.03 [0.99-1.07]	0.19	1.08 [1.01-1.15]	0.024	1.08 [0.99-1.18]	0.061
Combined	1.04 [1.00-1.09]	0.051	1.03 [0.99-1.06]	0.15	1.06 [1.01-1.12]	0.016	1.06 [1.00-1.12]	0.045

Adjusted for age, BMI, type 2 diabetes, SSB intake (drinks/day), alcohol intake (drinks/week), eGFR, hypertension and prescription of lipid-lowering medication. The Polynesian data are also adjusted by number of self-reported Polynesian grandparents. The combined analysis was additionally adjusted by ancestral group. NU: normouricaemia; HU: hyperuricaemia; OR: odds ratio; CI: confidence interval; Tg: triglyceride; FPLC: fast protein liquid chromatography; VLDL: very low-density lipoprotein; apo B: apolipoprotein B; BMI, body mass index; SSB: sugar-sweetened beverage; eGRF: estimated glomerular filtration rate.

**Table 4:** **Analysis for association of gout risk with lipid and apo B-associated traits in non-tophaceous gout (left) and gout excluding participants taking fenofibrate and statins (right)**

	**HU vs non-tophaceous gout**		**HU vs lipid-lowering excluded gout**	
	**Adjusted OR [95% CI]**	***P***	**Adjusted OR [95% CI]**	***P***
**Total Tg (mmol/L)**				
Europeans	1.11 [0.55-2.26]	0.77	1.30 [0.367-2.51]	0.44
Polynesians	0.95 [0.50-1.80]	0.88	1.16 [0.68-1.96]	0.59
Combined	1.04 [0.72-1.50]	0.82	1.24 [0.86-1.78]	0.25
**Total FPLC Tg (mmol/L)**				
Europeans	3.64 [0.96-13.82]	0.068	7.41 [1.42-38.56]	0.017
Polynesians	1.05 [0.29-3.77]	0.94	1.58 [0.62-4.05]	0.34
Combined	1.78 [0.91-3.48]	0.091	2.37 [1.17-4.83]	0.017
**VLDL Tg (mmol/L)**				
Europeans	5.10 [0.97-26.81]	0.054	26.15 [2.48-276.04]	0.007
Polynesians	1.72 [0.37-7.96]	0.49	3.18 [0.84-12.05]	0.089
Combined	2.78 [1.13-6.79]	0.025	4.77 [1.76-12.96]	0.002
**Total apo B (** **μmol/L)**				
Europeans	4.32 [0.86-21.69]	0.075	3.17 [0.71-14.09]	0.13
Polynesians	158.22 [1.95-1286.5]	0.024	7.23 [0.28-189.08]	0.24
Combined	11.58 [2.62-51.15]	0.001	4.32 [1.17-15.93]	0.028
**VLDL apo B (0.1** **μmol/L)**				
Europeans	1.99 [0.67-5.91]	0.22	1.85 [0.52-6.57]	0.34
Polynesians	0.39 [0.04-3.82]	0.42	1.80 [0.50-6.49]	0.37
Combined	1.32 [0.63-2.77]	0.46	1.64 [0.74-3.63]	0.23
**VLDL Tg:VLDL apo B**				
Europeans	1.06 [0.93-1.21]	0.39	1.05 [0.92-1.20]	0.43
Polynesians	1.48 [1.02-2.15]	0.041	1.11 [1.00-1.24]	0.060
Combined	1.07 [1.01-1.15]	0.029	1.07 [1.00-1.14]	0.042

Adjusted for age, BMI, type 2 diabetes, SSB intake (drinks/day), alcohol intake (drinks/week), eGFR, hypertension and prescription of lipid-lowering medication for the non-tophaceous analysis. The Polynesian data are also adjusted by number of self-reported Polynesian grandparents. The combined analysis was additionally adjusted by ancestral group. HU: hyperuricaemia; OR: odds ratio; CI: confidence interval; Tg: triglyceride; FPLC: fast protein liquid chromatography; VLDL: very low-density lipoprotein; apo B: apolipoprotein B; BMI, body mass index; SSB: sugar-sweetened beverage; eGRF: estimated glomerular filtration rate.

We next investigated apo B. It was notable that total apo B was significantly lower in HU than in NU in both ancestral groups (*P*_European_ = 0.036, *P*_Polynesian_ = 0.026) (Table 2). Given also the association in Europeans with serum urate of the *A1CF* gene [9], which encodes a complementation factor for the apolipoprotein B mRNA-editing enzyme catalytic polypeptide 1 [16], we tested the hypothesis that apo B is associated with HU by comparing total serum apo B levels between NU and combined HU/gout groups (Table 5). Each unit increase (μmol/L) in total circulating apo B was significantly associated with reduced risk of HU in Polynesians (OR = 0.20 [0.04 to 0.96], *P* = 0.044) but not in Europeans (OR = 0.46 [0.15 to 1.46], *P* = 0.19) with trending evidence for a protective role in HU in the combined sample sets (OR = 0.47 [0.21 to 1.04], *P* = 0.062). In contrast, each 100 nmol/L increase in VLDL apo B was not associated with an increased risk of HU in Europeans (OR = 4.04 [0.82 to 19.83], *P* = 0.086) or Polynesians (OR = 1.81 [0.51 to 6.47], *P* = 0.36) although there was evidence for increased risk of HU in the combined sample sets (OR = 2.50 [1.00 to 6.26], *P* = 0.050). There was, however, by linear regression analysis no association of apo B with urate levels in the combined NU and HU groups (Table 5). We also tested for a role of total circulating apo B in gout compared to HU (Table 3), observing positive association with gout (OR = 5.60 [1.73 to 18.18], *P* = 0.004 per μmol/L unit change) although there was no evidence for association of VLDL apo B (OR = 1.57 [0.80 to 3.09], *P* = 0.19 per 100 nmol/L unit change). Thus apo B negatively correlated with HU, but positively correlated with gout in the presence of HU.

**Table 5:** **Association analysis of apo B with hyperuricaemia (left) and serum urate (right)**

	**NU vs. HU and gout**				**Vs. urate in NU and HU groups**			
	**Unadjusted OR [95% CI]**	***P***	**Adjusted OR [95% CI]**	***P***	**Unadjusted β** **[95% CI]**	***P***	**Adjusted β** **[95% CI]**	***P***
**Total apo B (** **μmol/L)**								
Europeans	0.58 [0.24-1.39]	0.22	0.46 [0.15-1.46]	0.19	-0.026 [-0.071-0.019]	0.26	-0.014 [-0.048-0.020]	0.41
Polynesians	0.27 [0.10-0.75]	0.012	0.20 [0.04-0.96]	0.044	-0.038 [-0.071--0.004]	0.031	-0.017 [-0.057-0.023]	0.40
Combined	0.41 [0.22-0.77]	0.005	0.47 [0.21-1.04]	0.062	-0.034 [-0.061--0.007]	0.013	-0.020 [-0.045-0.005]	0.12
**VLDL apo B (0.1** **μmol/L)**								
Europeans	8.13 [2.04-32.42]	0.003	4.04 [0.82-19.83]	0.086	0.046 [0.011-0.081]	0.011	0.001 [-0.002-0.004]	0.48
Polynesians	2.32 [0.89-6.05]	0.084	1.81 [0.51-6.47]	0.36	0.045 [-0.007-0.098]	0.089	-0.002 [-0.004-0.0004]	0.099
Combined	3.80 [1.74-8.33]	0.001	2.50 [1.00-6.26]	0.050	0.046 [0.017-0.075]	0.002	-0.001 [-0.003-0.0005]	0.18

Adjusted for age, BMI, type 2 diabetes, SSB intake (drinks/day), alcohol intake (drinks/week), eGFR, hypertension and prescription of lipid-lowering medication. The Polynesian data are also adjusted by number of self-reported Polynesian grandparents. The combined analysis was additionally adjusted by ancestral group. NU: normouricaemia; HU: hyperuricaemia; OR: odds ratio; CI: confidence interval; apo B: apolipoprotein B; VLDL: very low-density lipoprotein; BMI, body mass index; SSB: sugar-sweetened beverage; eGRF: estimated glomerular filtration rate.

Given the organised chronic inflammatory nature of the tophus, with inflammatory factors that may promote resolution of gout attacks [17] and thus create a different inflammatory situation in acute tophaceous gout, we repeated the Table 3 analysis, but with tophaceous cases excluded (Table 4). Despite the exclusion of 53% of the Polynesian and 14% of the European cases, the significance of the total apo B and VLDL Tg: VLDL Apo B ratio analyses in the previous HU compared to gout analysis (Table 3) were strengthened (OR = 11.58, *P* = 0.001 compared with OR = 5.60, *P* = 0.004 and OR = 1.07, *P* = 0.029 compared to OR = 1.06, *P* = 0.005 respectively).

## Discussion

We observed increased levels of VLDL Tg in gout compared to asymptomatic HU. This increase was suspected to be driven by overproduction of VLDL particles in HU particularly in Europeans (as evidenced by a significantly higher VLDL apo B to total apo B ratio in HU and gout; Table 2) and additionally by the Tg-enrichment of existing VLDL particles (a significantly higher VLDL Tg to VLDL apo B ratio in gout; Table 2) in the Polynesian participants only. The association of each mmol/L increase in VLDL Tg with risk of gout in the presence of HU was restricted to Europeans (OR = 7.61, *P* = 0.011), although there was a trend towards increased risk in Polynesian participants (OR = 2.84, *P* = 0.069). We also associated reduced total apo B with HU and increased total apo B with gout compared to HU. Given this is a cross-sectional observational study, these results cannot ascribe causality to VLDL Tg and/or apo B in HU and in gout compared to HU. However the data, when considered with the previous literature, support further investigation of the role of VLDL Tg and/or apo B in HU and gout.

Apo B has been linked with downregulation of the innate immune system and the resolution phase of acute gout. When coating MSU crystals apo B suppresses neutrophil activation [5] (which is needed for endocytosis and lysis of these crystals in gout [18]). This is consistent with the clinical observation that apo B coats MSU crystals only when inflammation subsides in gout [19]. Apo B possesses domains enriched in positively charged amino acids, which have high affinity for binding to polyanionic molecules such as MSU crystals [20]. Our data, however, that associate increased levels of apo B with gout compared to HU are not necessarily inconsistent with the previous experimental evidence. It is possible that increased circulating apo B correlate with reduced synovial apo B in intercritical gout (all our participants were intercritical), which would reduce the suppression of innate immunity and increase the chance of acute gout. This hypothesis could be tested by measuring apo B and VLDL apo B in synovial fluid in NU, HU and gout. The maintenance of the association of apo B with gout compared to HU when tophaceous cases were excluded suggests heterogeneity and that future studies investigating a possible role for apo B in gout should stratify into tophaceous vs non-tophaceous gout. Recently a male gout patient with mitochondriopathy in Kearns-Sayre syndrome (KSS) was described [21]. This person had large MSU crystals that were less immunogenic and that exhibited little synergy with toll-like receptor 2 agonists, a response that was hypothesised to be due to different crystal conformational or chemicophysical properties. It would be interesting to evaluate the lipoprotein profile of MSU crystals from the KSS patient compared to non-syndromic gout patients.

Reduced total apo B was associated with HU whereas increased total apo B was associated with gout compared to HU. These observations suggest pleiotropic effects of apo B in gout. Previously, serum urate control in Europeans has been associated with genetic variation in the *A1CF* gene [9], which encodes a complementation factor for the apo B mRNA-editing enzyme catalytic polypeptide 1 (APOBEC). This enzyme is part of a complex that edits a translational stop codon within the apo B mRNA, causing synthesis of apo B-48 (present on chylomicrons) instead of the full-length apo B-100. In humans, this editing takes place in the intestine whereas apo B-100 (present on VLDL and LDL) is exclusively produced by the liver [22]. While it has not been confirmed if *A1CF* is the causal gene at this locus, the maximally associated genetic variants at this locus all map within *A1CF* [9] suggesting that this gene does control urate levels. The genetic data are consistent with our association of reduced total apo B levels with HU and lead to the prediction that the urate-increasing *A1CF* allele would functionally associate with reduced apo B. Collectively these data raise the possibility that reduced apo B is a risk factor for HU, yet increased VLDL Tg (with a single apo B molecule per VLDL particle) could be involved in gout in the presence of HU.

The fibric acid derivative fenofibrate is used in hypertriglyceridaemia to reduce VLDL, LDL and total triglycerides. It activates peroxisome proliferator-activated receptor alpha, which in turns activates lipoprotein lipase to reduce circulating Tg-rich particles [23]. In primary hypertriglyceridaemia fenofibrate reduces serum urate by increasing renal uric acid excretion via inhibition of urate transporter 1 [24,25] and reduces Tg levels without changing serum apo B levels [26]. Interestingly, the established serum urate-increasing response to an acute fructose load (ref [27] and refs therein) is negated by fenofibrate [26], suggesting a link with carbohydrate metabolism. Fenofibrate is not used as a primary management tool in gout, although its use is associated with maintenance of normouricaemia after withdrawal of frontline urate-lowering therapy in gout [28] and small trials show efficacy as an adjunct treatment for gout [29-31]. Our data, demonstrating a possible role for VLDL in gout compared to HU are consistent with previous evidence [26] supporting the use of fenofibrate as an adjunct treatment in gout with hypertriglyceridaemia.

One possible limitation of our study relates to the non-fasting status of the participants. This would have introduced additional variability into the Tg but not the apo B data - in a study of healthy subjects at four and eight hours postprandially, total apo B did not differ whereas total Tg levels increased when compared to fasting [32]. It is important to point out, however, non-fasting Tg and other lipids are better predictors of cardiovascular disease [33], supporting the use of non-fasting measures in studying metabolic risk.

Consideration of genetic data does support the hypothesis that aspects of Tg metabolism play a causal role in gout. For example, there are loci associated with control of serum urate levels that contain genes that are also associated with serum Tg levels (*GCKR, MLXIPL, A1CF*) [9] and gout [9,10]. A Mendelian randomization study, examining the relationship between the genetically attributable fractions of urate and triglyceride, provides evidence for a causal role of triglycerides in urate control but not the reverse [34]. These genetic data and the observational data presented herein support further testing of the hypothesis that apo B and VLDLTg play a causal role in gout. For example, in large sample sets genetic variants associated with apo B and VLDL Tg could be tested for association with gout compared to HU controls.

## Conclusions

We report association of reduced total apo B with HU, but increased apo B and VLDL Tg with gout compared to HU. These data support the hypothesis that VLDL metabolism plays a role in gout, with apo B having pleiotropic effects.

## Abbreviations

apo B: apolipoprotein B
BMI: body mass index
CHOD: cholesterol oxidase
eGFR: estimated glomerular filtration rate
ELISA: enzyme-linked immunosorbant assay
FPLC: fast protein liquid chromatography
GPO: glycerol-3-phosphate oxidase
HDL: high-density lipoprotein
HU: hyperuricaemia
IgG: immunoglobulin G
KSS: Kearns-Sayre syndrome
LDL: low-density lipoprotein
MSU: monosodium urate
NU: normouricaemia
NZ: New Zealand
OR: odds ratio
PAP: phenol and aminophenazone
Tg: triglyceride
VLDL: very low-density lipoprotein
